# Comparison of laparoscopic peritoneal vaginoplasty and sigmoid colon vaginoplasty performed during radical surgery for primary vaginal carcinoma

**DOI:** 10.1186/1477-7819-12-302

**Published:** 2014-09-30

**Authors:** Fengqiu Yao, Weidong Zhao, Gang Chen, Aijun Zhang, Fanglin Sun, Weiping Hu, Bin Ling

**Affiliations:** Department of Obstetrics and Gynecology, Anhui Provincial Hospital Affiliated with Anhui Medical University, 17 Lujiang Road, Hefei, 230001 China

**Keywords:** Laparoscopy, Primary vaginal carcinoma, Radical surgery, Vaginoplasty, Peritoneum, Sigmoid colon

## Abstract

**Background:**

Radical surgery of primary vaginal carcinoma typically involves partial or complete resection of the vagina, and young patients in particular can experience sexual dysfunction after surgery. Vaginoplasty is mandatory for this population, multiple vaginal reconstructive techniques have been reported. Here we attempted to determine whether the peritoneum is a feasible alternative to the sigmoid colon in vaginoplasty performed during radical surgery.

**Methods:**

Between February 2005 and July 2009, 12 patients underwent radical surgery for Federation of International Gynecology and Obstetrics Stage I primary vaginal carcinoma in the upper one-third of the vagina. To retain a sex life, the patients received vaginoplasty either with the peritoneum (peritoneal group, 5 patients) or with the sigmoid colon (sigmoid group, 7 patients) during radical surgery. Surgeries were performed at the Anhui Provincial Hospital in China. The data between the two groups was retrospectively analyzed.

**Results:**

The operating time was shorter for the peritoneal group than for the sigmoid group (*P* < 0.05). There were no significant differences in blood loss as well as in the length or width of the neo-vaginas between the two groups during surgery (*P* > 0.05). No metastasis or operation-related complications were observed in any of the patients. Six months after surgery, the neo-vaginas of both groups were smooth, soft, and moist. The neo-vaginas in the sigmoid group were similar in size during and 6 months after surgery. The neo-vaginas in the peritoneal group were shorter (although no less wide) 6 months after surgery (*P* < 0.05); length and width (that admitted two fingers) remained stable thereafter. All patients experienced a satisfactory sex life after surgery. Colposcopy revealed a good vaginal surface covered with squamous epithelium in the neo-vaginas of the peritoneal group, and intestinalization in the neo-vaginas of the sigmoid group. At the 36-month follow-up, all patients were clinically free of disease.

**Conclusions:**

Laparoscopic vaginoplasty using the peritoneum compared with using the sigmoid colon is simpler and more feasible for management of Stage I primary vaginal carcinoma. Its benefits include shorter operating time, no bowel disturbance, and production of a hygienic vaginal environment, as well as a potential sex life and oncologic outcome comparable to that of sigmoid colon vaginoplasty.

## Background

Primary vaginal carcinoma is a rare disease, representing 2% to 3% of all gynecological malignancies [1]. Although mainly observed in elderly women, approximately 30% of women with this disease are younger than 60 years of age [2]. Because of its rarity, there is no consensus on the optimum therapy, which must be individualized according to the patient’s medical condition, age, and general health, and the site and stage of the disease [3]. Although the standard treatment for all stages of primary vaginal carcinoma is radiation, some reports have shown better survival outcomes with surgery in patients with early-stage disease, especially young women [4, 5]. Although radical surgery consisting of radical vaginectomy and systematic dissection of lymphatic tumor drainage is a valid option [4], it typically involves partial or complete resection of the vagina, and young patients in particular can experience severe depression and sexual dysfunction after surgery. Vaginoplasty, particularly the creation of a neo-vagina, is mandatory for this population [6, 7].

Multiple vaginal reconstructive techniques including bowel flaps, skin grafts, peritoneal grafts, and musculofasciocutaneous flaps have been reported [8]. The choice of technique is important for obtaining functional and aesthetic results. Sigmoid colons are routinely used in vaginal reconstructions because of their unique characteristics including their close proximity to the operative site, their morphology, and their capacity to replicate the function of the vagina and early coitus [9, 10]. In 1974, Davydov and Zhvitiashvili [11] proposed a method for reconstructing the lining of neo-vaginas with the peritoneum from the Douglas pouch. To date, this technique has been used to form neo-vaginas laparoscopically, followed by surgery for congenital vaginal abnormalities [12–15].

In 2003, our department observed favorable survival and quality of life in patients with early-stage primary vaginal cancer who underwent vaginal reconstruction using the sigmoid colon [16]. However, because of bowel disturbance and the risks associated with this procedure (for example, excessive secretion by the neo-vagina, fistula, infection, and development of mucinous adenocarcinoma) [10], we attempted to determine whether vaginoplasty using the peritoneum is a feasible alternative.

## Methods

### Ethics Statement

The study was reviewed and approved by the ethics review board of Anhui Provincial Hospital. Written consent was obtained from each patient.

### Patients

Between February 2005 and July 2009, 12 patients between 39 and 61 years of age were referred to our department because of incidental findings, vaginal discharge, or irregular bleeding. The clinical characteristics of these patients are shown in Table 1. The two groups were comparable in terms of age, histological diagnosis, and tumor site, size, stage, and grade. None of the patients had extravaginal disease as determined via a complete preoperative work-up consisting of a gynecologic examination, colposcopy, abdominal and pelvic computed tomography, and sigmoidoscopy. Stage I primary vaginal carcinomas were diagnosed in accordance with the definition of the Federation of International Gynecology and Obstetrics. Other criteria for surgery were good overall health, routine biochemical testing, and electrocardiogram observations within normal limits. Once these criteria were met, laparoscopic vaginal surgery, including laparoscopic radical hysterectomy with pelvic lymphadenectomy, partial vaginectomy, and vaginal reconstruction using the peritoneum or sigmoid colon, was proposed to and accepted by each patient and her sexual partner. Informed consent was obtained from each patient and her sexual partner about the risks of the surgery.

**Table 1:** **Clinical characteristics of the patients**

	Peritoneal group (n = 5)	Sigmoid group (n = 7)	***P*** value
Age (years)	49 ± 8	50 ± 11	*P* > 0.05
Tumor site	Upper 1/3	Upper 1/3	
Tumor size (cm)	0.8 ± 0.2	0.9 ± 0.2	*P* > 0.05
Histotype	Squamous	Squamous	
Grade	1	1	
Stage (Federation of International Gynecology and Obstetrics)	I	I	

### Surgical procedures

**Figure 1:**
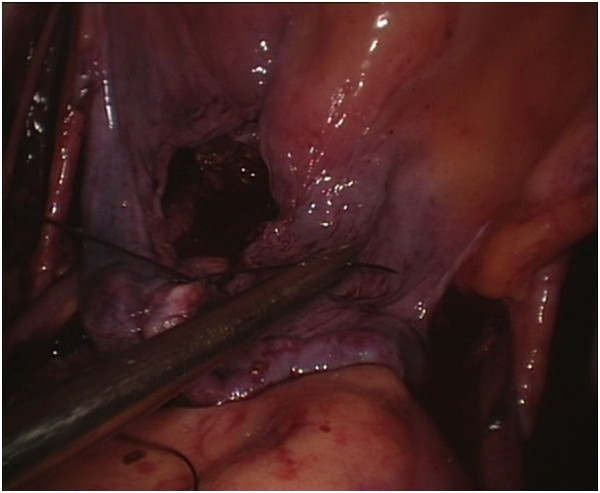
**After routine pelvic lymph node removal and radical hysterectomy were performed, the peritoneum covering the roof of the bladder, lateral pelvic side walls and Douglas pouch was preserved.**

**Figure 2:**
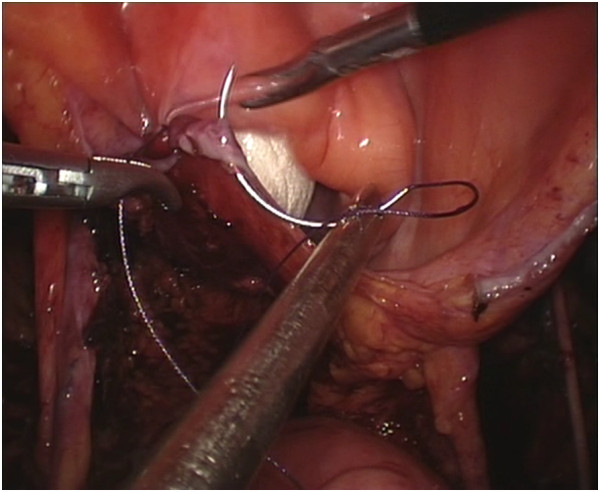
**A simple purse-string knot was tied by preserved peritoneum covering the roof of the bladder, lateral pelvic side walls and Douglas pouch.**

**Figure 3:**
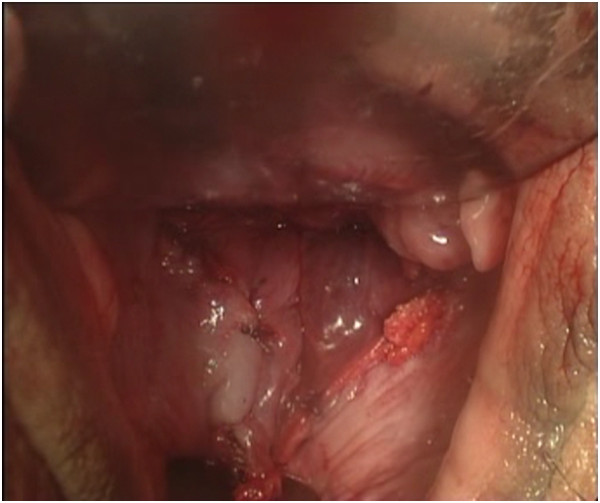
**The distal edge of the transplanted peritoneum was sutured to the margin of the vaginal stump intermittently.**

We performed the techniques for laparoscopic radical hysterectomy with pelvic lymphadenectomy, vaginectomy, and vaginoplasty using the sigmoid colon as published previously [16].The surgical procedures for the peritoneal group were as follows. The internal genitalia, peritoneum, and entire abdominopelvic cavity were carefully inspected after the installation of laparoscopic implements to rule out intraperitoneal tumor spreading and adhesion and to determine whether the patient’s anatomy was consistent with the preoperative assessment. During routine pelvic lymph node removal and radical hysterectomy, the peritoneum covering the roof of the bladder, the bilateral broad ligaments, the lateral wall of the pelvic cavity, and the Douglas pouch were preserved as much as possible (Figure 1). A partial vaginectomy was initiated by making a circumferential incision 3 cm below the lesion in the vagina. The anterior and posterior margins of the resected vagina were sutured together with consecutive stitches to prevent contamination from vaginal mucus and the carcinoma, and they were grasped and drawn forward using two Allis clamps at the 3 and 9 o’clock positions. The paravaginal tissues were divided along the vesicovaginal and rectovaginal spaces up to the bladder peritoneum fold and the rectouterine peritoneum fold, and the excised specimens were exteriorized vaginally. The reserved edges of the two round ligaments were sutured to the corresponding lateral margins of the vaginal excision to provide added support for the newly created vaginal vault using a 2–0 vicryl suture. The bottom of the reserved peritoneum covering the roof of the bladder, the lateral pelvic sidewalls, and the Douglas pouch were grasped and sequentially drawn forward through the vaginal canal, and the distal edge of the peritoneum was intermittently sutured to the margin of the vaginal stump. The transplanted peritoneum formed a canal that resembled a sleeve covering the lining of the new vagina (Figure 2). The proximal edge of the transplanted peritoneum (including the roof of the bladder, the lateral pelvic sidewalls, and the Douglas pouch) was tied with a simple purse-string knot using a 2–0 vicryl suture that was placed approximately 11 to 13 cm away from the vaginal orifice, creating a neo-vaginal vault separated from the bottom of the pelvic cavity (Figure 3). At the conclusion of the two operations, eight strips of iodoform gauze were inserted into the neo-vagina, and a single suction drain was positioned in the region of each pelvic sidewall via each lower port.

### Postoperative care

Routine postoperative care included perineal douching twice a day, treatment with antibiotics to prevent infection, and venous nutrition until the intestinal tract functionally recovered. Five days after surgery, the gauze and suction drains were removed. A special mold (9 cm in length, 3 cm in diameter) was inserted in patients in the peritoneal group for dilation (it was not required in the sigmoid group), and it remained in place for 6 months or until regular twice-weekly sexual intercourse resumed. Patients received a voiding trial approximately 1 week after surgery, and bladder dysfunction was defined as more than 100 ml of residual urine over 14 days.

Intraoperative and postoperative data were collected prospectively. All patients were asked to fill out the Female Sexual Function Index to evaluate sexual function 6 months after surgery; a score ≥24 indicates a satisfactory sex life [17]. All patients were followed-up by our team on a monthly basis for 3 years. Follow-ups included pelvic examination, a vaginal Papanicolaou test, colposcopy, and abdominal and pelvic computed tomography.

Statistical analysis was performed using SPSS software, version 15.0 (SPSS Inc, Chicago, IL). The Student *t-*test and the Mann-Whitney test were used to examine the differences between the groups. Statistical significance was defined as *P* < 0.05.

## Results

All laparoscopic-vaginal operations were accomplished successfully, and none were converted to laparotomies. Table 2 shows the intraoperative factors for both groups. The invasive depth of the tumor did not extend histologically beyond the vaginal wall in any of the patients. No metastasis was found on the incised margins of the parametrium, vagina, or any of the removed nodes. No patients received postoperative treatment apart from the routine postoperative care; no patient exhibited bladder dysfunction. Two patients experienced small superficial separations 8 days after surgery; the distal peritoneum was sutured to the margin of the residual vaginal mucosa, and the separations healed completely after 1 month without additional treatment.

**Table 2:** **Intraoperative factors between two groups**

	Peritoneal group (n = 5)	Sigmoid group (n = 7)	***P*** value
Operative time (minutes)	245 ± 46.4	300 ± 26.5	*P* < 0.05
Blood loss (ml)	320 ± 103.7	340 ± 82.7	*P* > 0.05
Metastasis	No	No	
Complications	No	No	
Length of vagina (cm)	12 ± 1.0	13 ± 1.4	*P* > 0.05
Pathology of surgical margin	Negative	Negative	
Depth of tumor (cm)	0.5 ± 0.2	0.6 ± 0.1	*P* > 0.05

After 6 months, the neo-vaginal walls of the patients in both groups were smooth, soft, and moist. Comparison of the length and width of the neo-vaginas during surgery and 6 months after surgery showed no change in the sigmoid group, while reduced length but not width was observed in the peritoneal group. The length of the neo-vaginas in the peritoneal group remained stable thereafter; however, the neo-vaginas were shorter in the peritoneal group than in the sigmoid group (*P* < 0.05). First intercourse after surgery occurred sooner in the sigmoid group than in the peritoneal group (*P* < 0.05). All patients eventually resumed satisfactory sexual lives, although the patients in the sigmoid group had some complaints of excessive mucus secretion.

**Figure 4:**
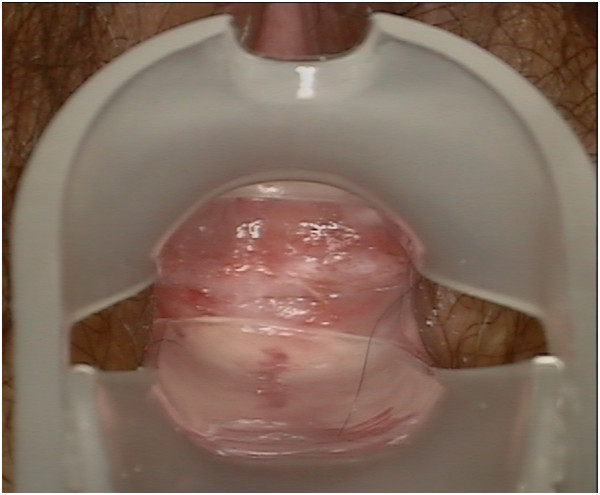
**At 6-month follow-up, colposcopy revealed a good vaginal surface covered with squamous epithelium in the neo-vagina of the peritoneal group.**

**Figure 5:**
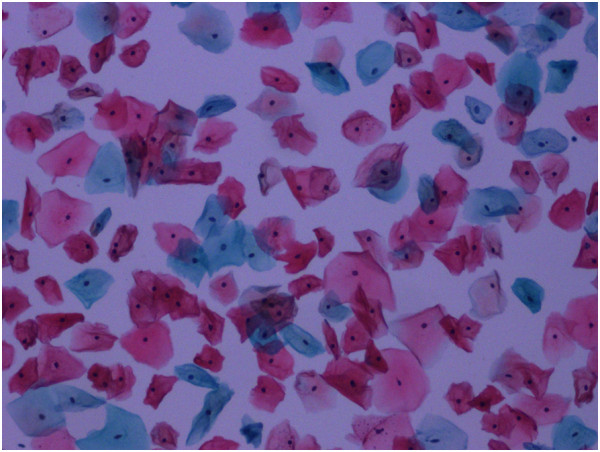
**Vaginal squamous cells such as those that occur naturally were detected with liquid-based cytology in the neo-vagina of the peritoneal group at 6-month follow-up.**

Table 3 shows the postoperative morbidity and treatment outcomes after 6 months. Colposcopy revealed good vaginal surfaces covered with squamous epithelium in the peritoneal group (Figure 4), while intestinalization was previously noted in the neo-vaginas of the sigmoid group [16]. Vaginal squamous cells such as those that occur naturally were detected via liquid-based cytology in the neo-vaginas of the peritoneal group (Figure 5). At the 36-month follow-up, all patients were clinically free of disease.

**Table 3:** **Postoperative morbidity and treatment outcomes after 6 months**

	Peritoneal group (n = 5)	Sigmoid group (n = 7)	***P*** value
Length of vagina (cm)	8.8 ± 0.8	12.5 ± 1.5	*P* < 0.05
Excessive secretion (n)	0	7	*P* < 0.05
Prolonged dilation (n)	5	0	*P* < 0.05
Time of first intercourse (months)	5.5 ± 0.3	3.5 ± 0.4	*P* < 0.05
Satisfactory sex life (n)	5	7	*P* > 0.05
Disease-free survival (n)	5	7	*P* = 1.00

## Discussion and conclusions

Cutillo and colleagues [18] described a conservative treatment for retaining sexual function after a vaginectomy (radical tumorectomy with pelvic lymphadenectomy), and Peters and colleagues [19] proposed a limited treatment (partial or total vaginectomy without dissection of regional lymph nodes). However, the standard treatment favored by gynecologists for lesions limited to the upper one-third of the vagina is vaginectomy and pelvic lymphadenectomy with or without hysterectomy, because it provides adequate margins and sufficient tissue for resection.

For the psychological rehabilitation of women after radical vaginectomy, creation of a neo-vagina is mandatory, especially in young patients who hope to resume sexual activity. In our opinion, the main aims of vaginal reconstruction are to: (1) restore the structure of the vagina such that future sexual activity is possible; (2) rectify vaginal defects with tissues having a good blood supply to promote rapid local healing; and (3) employ a simple, safe, and effective surgical technique. In our series, no intraoperative or postoperative complications were observed. The criterion for anatomic success was a neo-vagina of length 6 cm or greater and allowing easy introduction of two fingers within 6 months after surgery, and functional success was defined as self-reported satisfactory sexual intercourse [20]. Considering these anatomic and functional outcomes, we demonstrated that sigmoidal vaginoplasty and peritoneal vaginoplasty were similarly successful.

The main problem we experienced with peritoneal vaginoplasty was regular shrinkage of the neo-vagina. In addition, two patients experienced small superficial separations 8 days postoperatively, which may reflect the tendency of the peritoneal lining to shrink or indicate the presence of a vaginal mold. Although uncomfortable, placement of a mold in the neo-vagina for 6 months after surgery was deemed critical for maintaining the length and width of the neo-vagina. At the 6-month follow-up, colposcopy revealed a good vaginal surface covered with squamous epithelium in the peritoneal group, and liquid-based cytology detected vaginal squamous cells morphologically similar to those occurring naturally. However, the average length of the neo-vagina in the peritoneal group 6 months after surgery was less (8 to 10 cm) than that during surgery (11 to 13 cm), although the width (that admitted two fingers) was unchanged. Assuming that the peritoneal lining tends to shrink and adhere to itself before squamous metaplasia occurs, we recommend that a mold remain in place for 6 months after surgery, after which intermittent vaginal dilation with regular sexual intercourse is encouraged.

The advantages of sigmoid reconstruction include the sufficient diameter and elasticity of the sigmoid colon, which negate the need for a mold during the postoperative period; because a mold was not inserted, the sigmoid group resumed intercourse sooner than the peritoneal group (*P* < 0.05). Peritoneal vaginoplasty requires a relatively large amount of the pelvic peritoneum, as well as the pelvic cavity wall. Pelvic adhesion may limit the success of the surgery; therefore, peritoneal vaginoplasty for patients with a history of pelvic surgery is cautioned, and perioperative informed consent should include a discussion of the possible need to convert to a vaginal reconstruction using the sigmoid colon or other graft source.

We believe that lack of complications and postoperative morbidity observed in the peritoneal group may be explained by a number of factors. First, assuming adequate excisional scope of the operation, the round ligaments and the peritoneum covering the roof of the bladder, the lateral pelvic sidewalls, and the Douglas pouch were preserved as much as possible. Moreover, extensive dissection of the pelvic peritoneum with a good blood supply was not crucial. Second, the reserved sections of the two round ligaments were sutured to the corresponding lateral margins of the vagina to provide added support for the neo-vaginal vault. Third, a simple purse-string knot tied to the roof of the bladder, lateral pelvic sidewalls, and the Douglas pouch created a neo-vaginal vault and separated the bottom of the pelvic cavity from the neo-vagina. Fourth, expert identification of the abdominopelvic anatomy and performance of the laparoscopic procedure and open surgery for gynecologic malignancies were not required.

Vaginal reconstruction after radical surgery for primary vaginal carcinoma often includes a high risk of tumor recurrence. In our series, all patients were clinically free of disease at their 36-month follow-up. This finding demonstrates the safety of peritoneal and sigmoid colon vaginoplasty during radical surgery; however, we believe that the number of cases was too small and the follow-up was too short to provide an accurate estimate of the recurrence-free survival rate. We consider our study to be a pilot investigation in which we obtained informed consent for an experimental therapy and strictly adhered to a follow-up program aimed at detecting recurring or additional tumors at early stages. We note that accurate selection of patients is important; patients should be young and have Stage I squamous carcinoma of the upper vagina without involvement of organs outside the vaginal wall or metastasis to the regional lymph nodes.

In our series, peritoneal and sigmoid colon vaginoplasty were similar in terms of allowing a satisfactory sexual life, safety during surgery, and prevention of tumor recurrence during the follow-up period. Laparoscopic vaginoplasty using the peritoneum appears to be an ideal option because it offers the following advantages: (1) relative ease of performance; (2) no bowel disturbance; (3) fewer risks compared with sigmoid colon vaginoplasty (for example, prolapse of the neo-vagina, fistula, infection, and development of a mucinous adenocarcinoma); and (4) satisfactory sexual intercourse, with smooth, soft, moist, and hygienic neo-vaginal walls and no excessive mucus secretion. Its disadvantages include the need a mold for a prolonged period before squamous metaplasia occurs.
